# “In-between orthorexia” profile: the co-occurrence of pathological and healthy orthorexia among male and female non-clinical adolescents

**DOI:** 10.1186/s40337-022-00673-z

**Published:** 2022-11-03

**Authors:** Ecem Yakın, Sahar Obeid, Feten Fekih-Romdhane, Michel Soufia, Toni Sawma, Serena Samaha, Mariam Mhanna, Reine Azzi, Anthony Mina, Souheil Hallit

**Affiliations:** 1 Centre d’Études et de Recherches en Psychopathologie et Psychologie de la Santé, Université de Toulouse-Jean Jaurès, UT2J, 5 allées Antonio Machado, 31058 Toulouse, France; 2 Social and Education Sciences Department, School of Arts and Sciences, Lebanese American University, Jbeil, Lebanon; 3 The Tunisian Center of Early Intervention in Psychosis, Department of Psychiatry “Ibn Omrane”, Razi Hospital, 2010 Manouba, Tunisia; 4 Faculty of Medicine of Tunis, Tunis El Manar University, Tunis, Tunisia; 5 School of Medicine and Medical Sciences, Holy Spirit University of Kaslik, P.O. Box 446, Jounieh, Lebanon; 6 Research Department, Psychiatric Hospital of the Cross, Jal Eddib, Lebanon

**Keywords:** Orthorexia, Typological study, Cluster analysis, Students, Adolescence, Lebanon

## Abstract

**Background:**

The profile of adolescents with orthorexic eating behaviors remains to be explored. This study is the first to explore the typology of Lebanese adolescents from a large non-clinical sample based on orthorexia nervosa (ON) and healthy orthorexia (HO).

**Method:**

A total of 555 adolescents (aged between 15 and 18 years) completed a set of questionnaires assessing orthorexic behaviors, self-esteem, stress, depressive and anxiety symptoms. Cluster analysis based on ON and HO scores was used to identify the typology of the sample. More precisely, this analysis was used to reveal and distinguish between naturally occurring subgroups of individuals with different orthorexic eating profiles, within the studied sample. Further, a series of one-way ANOVA was used to compare observed clusters based on their scores on used questionnaires. This analysis was used to capture the behavioral and psychological differences between previously yielded subgroups of individuals.

**Results:**

Cluster analysis based on ON and HO scores yielded 3 distinct groups: “Low orthorexia”, “Moderate in-between orthorexia” and “High in-between orthorexia”. While the first group represented individuals with no particular (healthy or pathological) interest in healthy eating, the two latter groups represented those with respectively moderate and high degrees of an interest in healthy eating that has both pathological and healthy aspects. Significant differences between clusters regarding their levels of stress, depression, anxiety and self-esteem was observed, yet they were found to be negligible due to poor effect sizes.

**Conclusion:**

Findings from this study suggest that ON and HO can indeed co-occur among adolescents, that this co-occurrence can be experienced at different severity levels. Low effect sizes for ANOVA comparisons may suggest the possibility of the co-occurrence of ON and HO reducing the negative effects of ON behavior to some degree. The potential role of confusion around what constitutes "healthy eating" in the emergence of these "in-between orthorexia" profiles is put forward.

## Plain English summary

The profile of adolescents with orthorexic eating behaviors remains to be explored. Lebanese adolescents in this study were categorized into three groups: “Low orthorexia”, “Moderate in-between orthorexia” and “High in-between orthorexia”. Significant differences between clusters regarding their levels of stress, depression, anxiety, and self-esteem was observed, yet they were found to be negligible due to poor effect sizes. Findings from this study suggest that ON and HO can indeed co-occur among adolescents and that this co-occurrence can be experienced at different severity levels.

## Introduction

Orthorexia nervosa (ON) has been suggested as a new eating disorder diagnosis, albeit without established consensus [1]; and has been defined as a pathological or obsessional preoccupation with perceived healthy nutrition leading to emotional and nutritional repercussions (e.g., anxiety, distress, weight loss, malnutrition), as well as psychosocial dysfunction [2].

More precisely, individuals with ON are thought to have unrealistic beliefs about the properties and the potential health benefits of food (i.e., believing that eating and/or avoiding several nutrients can help treat or avoid certain diseases) [3, 4], and therefore spend most of their time planning, purchasing, and eating “healthy” meals and avoiding or even eliminating entire categories of food perceived as unhealthy [5]. When their personal dietary rules are transgressed, individuals with ON are believed to experience excessive concern and guilt and to further engage in subsequent compensatory behaviors (e.g., intensifying restrictions, excessive exercice or ‘cleanses’ which reflects a highly restrictive and selective food and/or liquid consumption aiming to rid the body of substances perceived as unhealthy) [4, 6]. These behaviors are further suggested to result in significant physical, psychological, and social impairments [3, 4, 7]. Indeed, many studies reported that ON could result in significant weight loss, malnutrition, and other medical complications (e.g., bradycardia, metabolic acidosis etc.) [3, 4, 7], as well as disruption of social relationships and social isolation (i.e., hiding away, avoiding friends and family who do not share similar beliefs and eating patterns, refusing other’s opinions on diet) [8, 9].

ON has more recently been conceptualized as a clearly distinct construct from healthy orthorexia (HO), which refers to an “eating style” characterized by a non-pathological interest in eating right or healthy and "eating healthily as part of one’s identity" [10, 11]. Orthorexic eating behavior has thus been suggested to represent a bi-dimensional construct, including both pathological (ON) and non-pathological (HO) aspects of an interest towards healthy eating [12]. There is increasing evidence that ON and HO are conceptually distinguishable behaviors, (pathological and protective eating behaviors, respectively) that are differently related to psychopathology and outcomes [1, 10]).

More precisely, ON has been found to be associated with several important psychopathological constructs such as self-reported depression, having a diagnosis of major depressive disorder, suicidal thinking, history of mental illness, anxiety and all aspects of the Dark Triad (Machiavellianism, narcissism, and psychopathy) [12–16]. According to some recently conducted studies, ON also displays associations with other disorders including illness anxiety disorder, psychotic disorder [17], autism spectrum disorder [18], body dysmorphia [19], somatoform disorders [20] and exercise addiction [21]. Although the associations between HO and some of these psychopathological constructs remain to be explored to this date, existing data suggest that, in contrary to ON, HO is not (or even inversely) associated with measures of anxiety and depression, eating disorder symptoms (ED), obsessive–compulsive symptoms (OCD), concern over mistakes, negative affect, body-image dysphoria or health anxiety [22], stress or impulsivity [13], weight control and/or affect regulation related dietary choices, and with inadaptive personality traits (e.g., lower levels agreeableness) [10, 11, 23].

Many of the psychopathologies mentioned in above paragraph are known to onset in early adolescence. Indeed, there is mounting evidence suggesting that adolescent years represent a period of life characterized by heightened vulnerability to the development of anxiety disorders [24], depressive disorders [25], personality disorders (especially borderline pathology) [26] and eating disorders [27, 28]. Likewise, several empirical studies focusing specifically on adolescent populations in orthorexia literature, have obtained results suggesting that this age group could also be at risk for the development of ON. For instance, one study, which is to our knowledge the first to assess ON among adolescents, reported that ON prevalence among Polish adolescents would be similar to that recorded in adult populations [29]. According to another study [30] Polish students of junior secondary school would be more at risk of ON compared to students of senior secondary school, due to their great interest in physical attractiveness and higher BMI. A study addressing ON among Turkish adolescents, reported an association between the deterioration of eating attitudes and increased ON tendency [31]. Another study [32] confirmed the presence of orthorexic tendencies among Turkish adolescent populations and highlighted the greater presence of ON tendency among adolescents with Diabetes Mellitus Type 1 diagnosis compared to healthy controls. A recent study, which is to our knowledge the first to address both ON and HO behaviors among Lebanese adolescents [33], reported higher ON scores in females compared to males, as well as a negative correlation between age and ON scores, which can further indicate that younger adolescents being more vulnerable to the development of pathological orthorexia compared to other youth groups with slightly older age. Finally, a recently conducted literature review [34] suggested that adolescents, similar to young adults, could be at risk to the development of ON, with a particular emphasis given to the shared psychological features and risk factors (e.g., negative body image, weight pressures, and disordered eating behaviors) between EDs which tend to occur during adolescence and ON, as one possible explanation.

Although very informative and important for our understanding of orthorexia phenomenon, the aforementioned studies focusing specifically on adolescent populations remains quite limited in number in the orthorexia literature which consists mostly of studies conducted with older populations (e.g., individuals over the age of 18) [33, 35]. However, adolescents represent a particularly vulnerable subgroup within young populations due to, inter alia, the developmental changes in neural and hormonal systems, the stress induced by increased academic social, and family demands [36], identity exploration, the strong desire to fit in and to be appreciated by peers [37]. These populations are also suggested to have greater denial when it comes to their disturbed eating patterns, less desire for help and less elimination of junk food from their diets [38] compared to older populations. The current state of research on orthorexia among adolescents can therefore benefit from further empirical evidence. Exploring the vulnerability towards orthorexia, and more precisely the characteristics and behavioral patterns of adolescents with greater ON and HO tendencies, could considerably extend our understanding of the onset, course and the outcomes of healthy and pathological orthorexia during this period of life. This could also help clinicians and researchers develop appropriate prevention, intervention, and education strategies for young populations.

Compared to variable-centered approach, person-based methods (i.e., cluster analysis, latent class, or latent profile analysis) can be more useful to better identify the vulnerability towards orthorexia during adolescent years, through the exploration of the profile and behavioral patterns of adolescents with ON and HO. In fact, variable-centered approaches (e.g., correlations, regressions, or SEM models) are commonly employed in the orthorexia literature, and they also represent the traditional and dominant approach in social sciences [39]. As the purpose of these methods is to explain relationships between variables of interest in a population (i.e., effects of one variable on another) [39–41], they allow researchers to better understand the associations and characteristics of orthorexia phenomenon. On the other hand, as they also focus on describing the average behavior of the sample they might underestimate the heterogeneity of individuals and display significant limitations when it comes to understand the behavior of a specific subgroup or a person [40–42]. In contrast, person-centered approaches are thought to overcome this reductionism as they focus on relationships among individuals, identify and distinguish between naturally occurring homogenous groups of individuals with similar characteristics or behavioral patterns [42–44]. It should also be noted that several studies suggested that traditional cluster analysis could provide more homogenous groups and more effectively distinguish between clusters, compared to some of the other person-based methods, such as Latent Class Analysis [45].

Despite their advantages over variable-centered approach, to our knowledge, only very few studies were conducted using person-centered methods) [46–50]. Additionally, among these latter, only one study, to our knowledge, assessed the distinctiveness between female adults with ON and those with HO behaviors [49]. This typological study [49], used cluster analysis and identified four naturally occurring distinct groups of individuals with different patterns of orthorexic eating behavior, labelled as follows: “Healthy orthorexia”, “Orthorexia Nervosa”, “Low-orthorexia” and “In-between orthorexia” clusters. According to authors [49], the “Healthy orthorexia” cluster represented individuals with a healthy interest in healthy eating, while the “Orthorexia Nervosa” group represented those with a pathological preoccupation with rigid healthy diet; the “Low-orthorexia” cluster represented individuals with no particular interest in healthy diet, whereas the last group called “In-between orthorexia”, characterized with high scores on both ON and HO, represented those who have both pathological and healthy attitudes and behaviors in regard to a “healthy” diet (i.e., engaging in self-punishment when their personal dietary rules are transgressed along with trying to convince people to follow a healthy eating). These groups also demonstrated significant differences regarding their eating behaviors (i.e., levels of intuitive eating) and psychopathological features (i.e., levels of anxiety, depression, and self-esteem). According to the authors [49], these results not only provided supportive evidence for the distinctiveness of ON and HO behaviors, but also suggested that both behaviors can be experienced together, or put differently, can co-occur among some individuals.

To our knowledge, there is no typological studies to date assessing healthy and pathological orthorexic behaviors in adolescent populations. As mentioned in above paragraphs, identifying, and distinguishing between the profiles and behavioral patterns of adolescent with ON and HO could have important implications in orthorexia research as well as in clinical settings. It could contribute to our understanding of the vulnerability towards orthorexia during this period of life and help clinicians better capture the heterogeneity of adolescent with orthorexic behaviors, to develop prevention and treatment techniques accordingly. It could also help researchers to clarify boundaries between ON and HO and arrive at a consensus definition and clear diagnostic criteria for the two constructs.

The aim of the current study was, therefore, (a) to identify the typology of adolescents of both genders aged between 15 and 18 from a large non-clinical sample based on their ON and HO behaviors using cluster analyses, and (b) to investigate whether observed profiles differed from each other regarding their levels of self-esteem, anxiety, depression, and stress. As there is neither former study nor model on the typology of adolescents aged between 15 and 18 years with ON and HO behaviors, we used an exploratory approach and avoided formulating specific hypothesis on clusters.

## Methods

### Study design

A total of 555 adolescents (15–18 years), currently residing in Lebanon, enrolled in this cross-sectional study (May–June 2020) using a proportionate random sample taken from all the Lebanese governorates (Beirut, Mount Lebanon, South, North and Bekaa). The COVID-19 pandemic outbreak in Lebanon forced a national lockdown, resulting in the closedown of schools. This situation imposed a snowball sampling technique through an electronic copy of the questionnaire on Google forms. The study’s main aims and goals, in addition to instructions for filling the questionnaire, were conveyed online for the participants, prior to their participation. In addition, there were no credits received for participation. A non-clinical adolescent sample was preferred as there is significant evidence that orthorexic behaviors are experienced by non-clinical young adult and adolescent populations [30, 34]. Furthermore, studying non-clinical populations also allows investigating the occurrence of several symptoms without complications of an established illness [51].

### Minimal sample size calculation

As per the G-power software [52], a minimal sample of 395 was required relying on an effect size f2 = 2%, an alpha error of 5%, a power of 80%, and 10 factors to be entered in the final model.

### Assessment measures

The questionnaire distributed to the participants was in Arabic. A pilot study on 15 adolescents was done to ensure that all questions are well understood. No revisions were done, therefore, the information remained in the final database. The first part of the questionnaire was comprised of questions evaluating the socio-demographic details of the participants (age, gender and residency governorate). The socioeconomic status (SES) of the family was reflected using the Household Crowding Index (HCI) that was computed by dividing the number of people living in the house by the number of rooms, in the household, while excluding the kitchen and bathrooms; higher HCI correlates with lower SES [53].

The second part of the questionnaire included the following measures and scales:

#### Teruel Orthorexia Scale (TOS)

Validated in Lebanon [33], this 17-item instrument assesses ON with two separate dimensions [12]: 9 items for Healthy Orthorexia or “HO” (e.g., “I mainly eat foods that I consider healthy”) and 8 items for Orthorexia Nervosa or “ON” (e.g., “Thoughts about healthy eating do not let me concentrate on other tasks”). Responses are provided on a four-point Likert scale ranging from 0 = strongly disagree to 3 = strongly agree. Scores by dimension were computed as the sum of the item responses. No specific timeframe was asked from the participants while responding. Higher scores would indicate higher orthorexia nervosa. In the current study, Cronbach’s alpha = 0.83 and 0.86 for the ON and HO subscales respectively.

#### The Rosenberg Self Esteem Scale (RSES)

The Rosenberg Self Esteem scale is a self-reported ten items questionnaire used to assess attitudes and beliefs regarding self-worth [54]. Higher scores indicate higher self-esteem (Cronbach’s alpha in the current study = 0.77). The Arabic version of the scale has been previously employed [55, 56].

#### Hamilton Anxiety Rating Scale (HAM‑A)

It is a widely used instrument in clinics and research for measuring the severity of anxiety symptom [57]. The scale was translated to Arabic and linguistically validated in the Lebanese population [58]. It comprises 14 items targeting both psychological and somatic symptom-defined elements. Each question scored on a basic numeric scoring of 0 (not present) to 4 (severe), with a total score ranging from 0 to 56, with higher scores delineating higher anxiety (Cronbach’s alpha in the current study = 0.89).

#### Patient Health Questionnaire (PHQ-9)

It’s a 9-item instrument [59] developed for making DSM-4 criteria based diagnoses of depressive disorders encountered in primary care and is validated in Lebanon [60]. Questions are about the level of interest in doing things, feeling down or depressed, difficulty with sleeping, energy levels, eating habits, self-perception, ability to concentrate, speed of functioning and thoughts of suicide. Each item is scored on a four-point Likert scale, ranging from 0 (not at all, several days, more than half the days) to 3 (nearly every day). The total sum of the responses suggests varying levels of depression (minimal, mild, moderate and severe) (Cronbach’s alpha in the current study = 0.83).

#### Beirut Distress Scale (BDS-10)

The Beirut Distress Scale, developed in Lebanon [61], was used to assess the intensity of psychological distress by using ten questions. The points range from 0 (never) to 3 (always), with higher scores indicating higher psychological distress (Cronbach’s alpha in the current study = 0.82).

### Statistical analysis

**Fig. 1:**
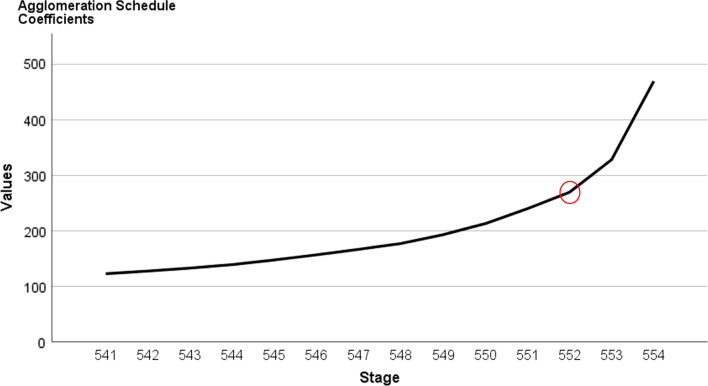
Hierarchical clustering: Scree plot of Agglomeration Schedule coefficients by stage. *Note.* The horizontal axis represents successive stages of hierarchical cluster analysis at which the similar clusters are merged. The vertical axis represents Euclidean distance coefficients of clusters being combined at each stage. The first noticeable increase in coefficient values is highlighted, confirming that a partition into three clusters was appropriate

The SPSS software version 23 [62] was used to conduct data analysis. No missing values were found since all questions were required [63]. Reliability using Cronbach’s alpha values for different scales were also included. Since the sample included more than 100 students [64], parametric tests were used. As the current study has an exploratory design, we first conducted a hierarchical cluster analysis based on the Z scores for ON and HO on the whole sample, using the Ward’s method with Euclidean distance. Ward’s method was suggested to be more appropriate for various types of data structures compared to other hierarchical algorithms [65], and the Euclidean distance, a commonly used distance measure, is known to be more suitable for numerical variables [66, 67]. Overall, the hierarchical cluster analysis is used to reveal the naturally occurring subgroups in the sample that are homogenous with regards to highly similar observations they contain, yet significantly different from each other [68]. The optimal number of clusters has been identified based on information from both agglomeration schedule and dendrogram. More precisely, the agglomeration schedule lists all the successive steps (N-1) that cluster analysis uses to progressively merge clusters with greatest similarity. It also provides coefficients to indicate the distance of the two clusters being combined at given stages. As the agglomeration schedule can be quite large, we used a scree plot to visually represent the changes in coefficient values at each step (Fig. 1). The first noticeable jump or increase in coefficient values between two consecutive stages displayed in the plot is considered as an optimal stopping point for clustering process; it indicates that clusters being merged are becoming more heterogeneous and that it would be ideal to stop the clustering at the given step before the clusters become too dissimilar from each other [66]. This suggested stopping point is then used to cut and interpret the dendrogram which illustrates the hierarchical clustering process [66, 67]. Detailed explanations on how information from both agglomeration schedule and dendrogram are incorporated to find the optimal number of clusters can be found in Yim and Ramdeem [66]. After the number of clusters have been identified, K-means clustering was used to assign each individual to the identified clusters. Cluster group differences regarding self-esteem, anxiety, depression and stress levels were tested using one-way ANOVA (Analysis of Variance) for each variable. The ANOVA test was used to compare three or more means. Bonferroni post hoc tests were conducted to the groups two by two. Partial eta squared (ɳ_p_2; representing the effect size) was also recorded. ɳ_p_2 values of 0.01, 0.06 and 0.14 indicate small, medium and large effects respectively [69] and *p* < 0.05 was considered significant.

## Results

The sociodemographic characteristics of the students are summarized in Table 1. Their mean age was 16.66 ± 1.00 years, with 24.3% males.

**Table 1:** Sociodemographic and other characteristics of the participants (N = 555)

Variable	N (%)
*Sex*	
Male	135 (24.3%)
Female	420 (75.7%)
*District*	
Beirut	62 (11.2%)
Mount Lebanon	332 (59.8%)
North	92 (16.6%)
South	29 (5.2%)
Bekaa	40 (7.2%)

	Mean ± SD
Age (in years)	16.66 ± 1.00
Household crowding index	0.99 ± 0.52

### Cluster analysis

**Fig. 2:**
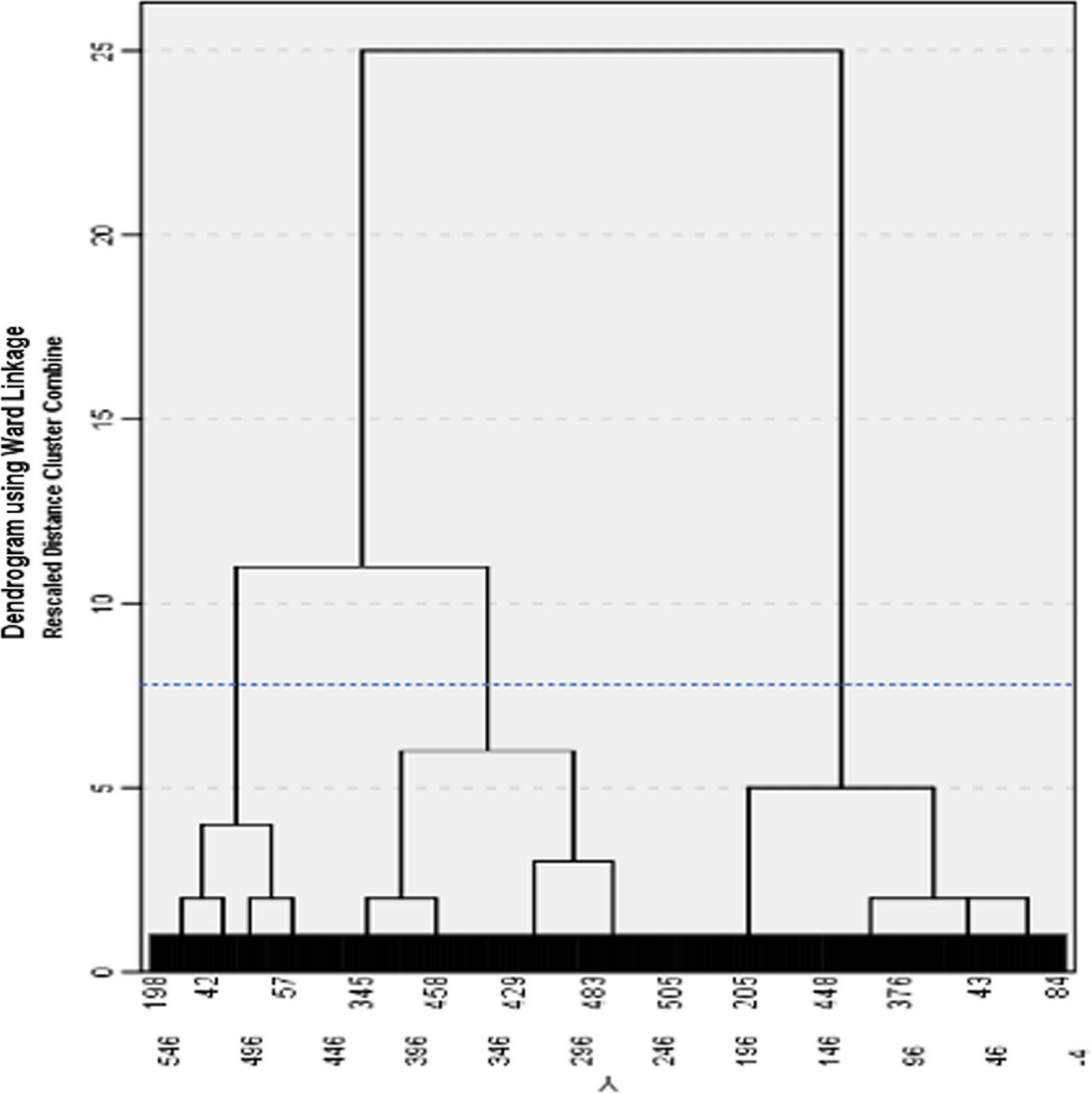
Hierarchical clustering: Dendrogram with added line indicating suggested stopping point. *Note.* The vertical axis represents the distance between clusters reflecting their dissimilarity, while the horizontal axis represents observations in the dataset, which form the clusters

The dendrogram and the agglomeration schedule were used to identify the most appropriate number of clusters [66]. In the agglomeration schedule, the first noticeable increase in coefficient values was observed between when three clusters were merged to two clusters, from 269.47 to 328.22 with a difference of approximately 58.75. This increase in agglomeration schedule, which is also presented in the scree plot (Fig. 1), indicated that the passage from three to two clusters would have more impact on the heterogeneity of the clusters than previous stages of the analysis. Therefore, the three-cluster solution was found to be the most appropriate. The dendrogram illustrating the hierarchical clustering process can be found in Fig. 2. A discriminant analysis showed clear differences between the four clusters (Wilk’s λ = 0.16, *p* < 0.001) with 95.0% of cases correctly classified.

**Fig. 3:**
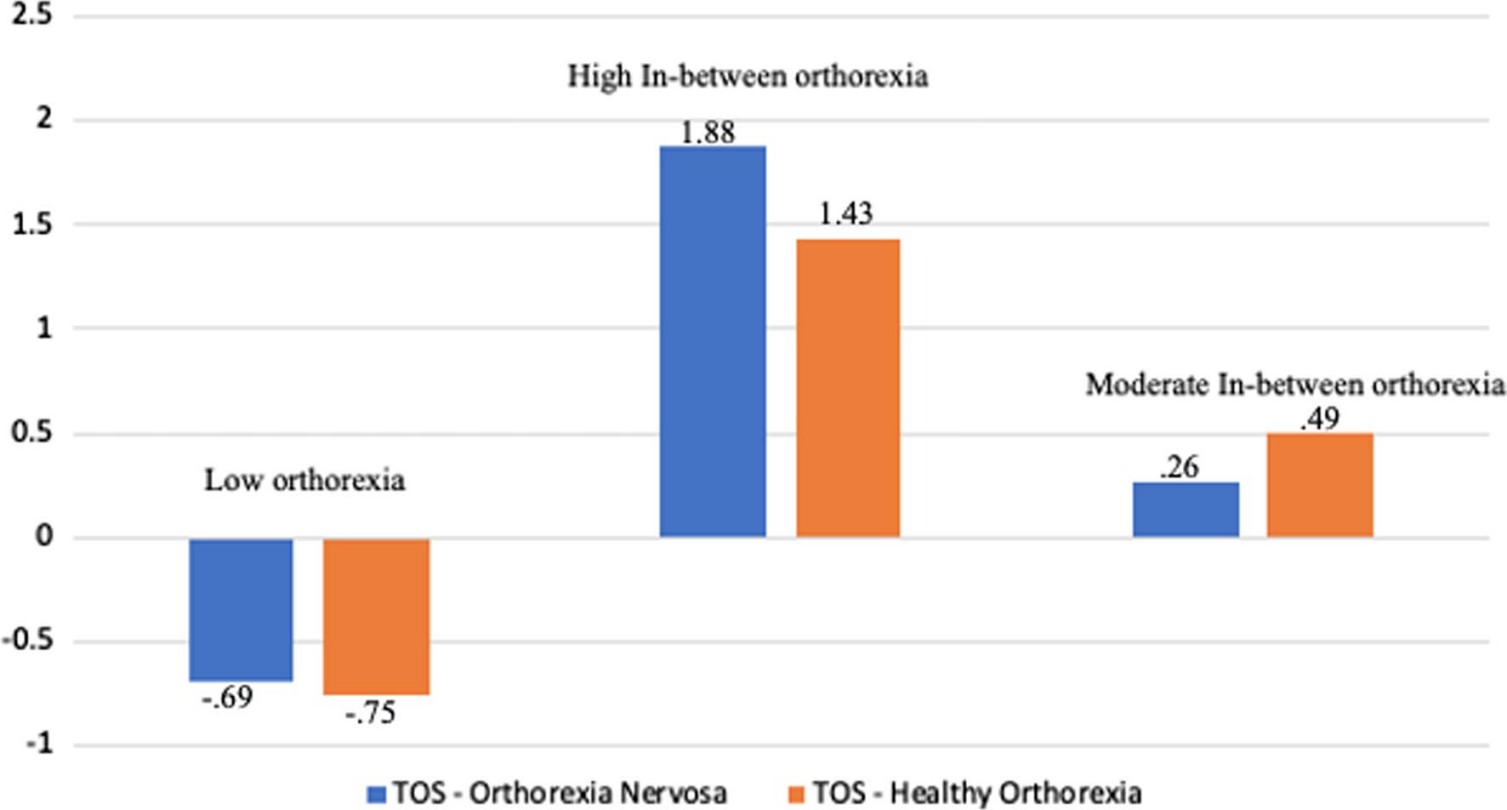
K-means clustering: Graph of means for the three-cluster solution based on standardized scores for Healthy Orthorexia and Orthorexia Nervosa

Data revealed a first group [n = 272 (49%)] characterized with low mean ON and HO scores that were below the sample means by more than half standard deviation (SD) for both scores. This group was labeled “Low orthorexia” (LO). The second group [n = 86 (15.5%)] had the highest mean ON and HO scores that were above the sample means by more than 1.5 SD for both scores; this cluster was called “High in-between orthorexia” (H-IBO). A third group [n = 197 (35.5%)] was characterized with moderate increase in the mean ON and HO scores that were both  above the sample means by less than 0.5 SD. Thus, this group was labelled “Moderate in-between orthorexia” (M-IBO) (Fig. 3).

### Comparison between the three clusters

Using one-way analysis of variance and Tukey’s HSD post-hoc tests (Table 2), we compared the three clusters based on self-esteem, stress, symptoms of anxiety and depression. Higher mean anxiety (17.93), depression (10.48), and distress (11.47) scores were significantly found in the “High in-between orthorexia” group, while a higher mean self-esteem score (18.10) was found in the “Low orthorexia” group (Table 2). The post-hoc comparison, comparing the groups two by two, revealed that a higher self-esteem mean score was significantly found in the “Low orthorexia” compared to the “High in-between orthorexia” group, whereas a higher depression mean score was significantly found in the “High in-between orthorexia” compared to the “Low orthorexia” group. Finally, higher anxiety and stress mean scores were found in the “High in-between orthorexia” compared to the “Low orthorexia” and “Moderate In-between orthorexia” groups.

**Table 2:** Typology of individuals based on Healthy Orthorexia and Orthorexia Nervosa

	“LO” cluster n = 272 (49%)	“H-IBO” cluster n = 86 (15.5%)	“M-IBO” cluster n = 197 (35.5%)	F	*p*	ɳ_p_2	Significant post-hoc comparisons
Self-esteem	18.10 (5.42)	16.45 (5.50)	17.31 (5.32)	3.39	0.034	0.012	LO > H-IBO
Anxiety	13.25 (10.14)	17.93 (10.67)	14.30 (10.47)	6.69	0.001	0.024	H-IBO > LO, M-IBO
Depression	8.35 (5.49)	10.48 (6.25)	8.89 (4.97)	4.98	0.007	0.018	H-IBO > LO
Stress	9.25 (5.57)	11.47 (6.02)	9.52 (5.39)	5.29	0.005	0.019	H-IBO > LO, M-IBO

Results are adjusted over age and gender

### Comparison between genders

A significantly higher percentage of females was found in the “Moderate In-between orthorexia” group, whereas a significantly higher percentage of males was found in the “Low orthorexia” group (Table 3).

**Table 3:** Comparison between genders in terms of clusters

	“LO” cluster n = 272 (49%)	“H-IBO” cluster n = 86 (15.5%)	“M-IBO” cluster n = 197 (35.5%)	Pearson Chi-square	*p*
*Gender*				9.232	0.010
Males	81 (60.0%)	19 (14.1%)	35 (25.9%)		
Females	191 (45.5%)	67 (16.0%)	162 (38.6%)		

## Discussion

Although there is a growing interest towards the identification of profile and behavioral patterns of individuals with orthorexic eating behavior [46–49], to our knowledge, there are no studies to date exploring the typology of adolescents based on their orthorexic eating behaviors. By investigating the typology of non-clinical male and female adolescents based on their ON and HO scores using cluster analysis, we believe that our research can help clarify boundaries between ON and HO and arrive at a consensus definition and clear diagnostic criteria for the two constructs.

In this study, cluster analysis yielded three distinct groups of individuals: “Low orthorexia” (LO), “High in-between orthorexia” (H-IBO) and “Moderate in-between orthorexia” (M-IBO). The first group called “LO” represented adolescents with no particular (healthy nor pathological) interest in healthy eating. The other two groups of adolescents labelled as “M-IBO” and “H-IBO” represents respectively moderate and high degrees of an interest in healthy eating that has both pathological and healthy aspects. This finding is in line with and further expands on previous data [49] on the existence of an “In-between orthorexia” profile in female adults, as it suggests that ON and HO can also co-occur among adolescents of both genders, and that this co-occurrence can be experienced in different severity levels. This would imply that among Lebanese adolescents, an "interest in healthy eating" does not fully evolves into a healthy eating behavior (HO), but also to some extent into problematic eating behavior (ON). For instance, these adolescents with “In-between orthorexia” profile may try to convince others to follow their healthy eating habits and not feel distracted by healthy eating related thoughts during their daily activities (HO); while they also feel overwhelmed and even punish themselves if they eat food that they consider unhealthy (ON).

Although additional research will be needed to further explore the reasons for this finding, in our opinion one potential explanation revolves around the confusion regarding what constitutes a healthy diet among young populations. More precisely, young populations are susceptible to developing eating disorders and orthorexic eating behaviors [27, 34, 70, 71]. This proneness has been attributed to, inter alia, socio-cultural influences and social media use among adolescent populations in eating disorders and orthorexia literature [72–75]. Recently, some authors [76] pointed out that the debate between scholars over the definition and categorization of ON among other mental disorders risk misinforming general populations, creating confusion about what represents pathological and non-pathological “healthy eating” patterns. Considering these issues, we argue that our findings suggesting the existence of adolescents with "in-between orthorexia" profile (engaging in a healthy eating which has both pathological and healthy aspects), reflect this confusion among adolescents over what constitutes a healthy diet. In fact, it is possible that these populations encounter social pressures and social media contents channeling information both related to pathological and healthy eating patterns, which may further increase their tendency towards both ON and HO, creating an “in-between” profile of orthorexia. Indeed, as several authors pointed out, adolescents and young adults may not necessarily have ability and knowledge “to draw a distinct line between healthy and disordered eating” patterns [34].

Furthermore, adolescents may have limited food choice for two main reasons. Time scarcity produced by the school demands make them less likely to make their own meals, and rather rely on home-made meals provided by their families. The other reason is that healthy foods may be not affordable due to financial constraints and parental financial monitoring. This lack of control over their food choices may explain in part why a distinct HO profile did not emerge in our sample. This calls for further research in young adult samples with more spare time and financial independency to confirm our assumptions.

Additionally, social media platforms represent potential influencers of adolescents’ dietary perceptions and habits [77, 78], especially given their easy accessibility, high usage among this age group, and high rates of eating-related and appearance content. This content may be harmful more than it is beneficial and negatively impact dietary habits [77, 79]. One example is the “Fitspiration” trend which promotes “healthy eating” through unrealistic "healthy body" ideals, excessive exercise and even unreliable health information, and which has been suggested to result in body dissatisfaction, negative mood, compulsive exercise, eating disorders and psychological distress [77, 79]. Such social media content may cause a misunderstanding or confusion around the distinction between healthy and unhealthy dietary patterns. Therefore, future research should explore the role of sociocultural factors, use of social media and adolescents' perceptions of "healthy eating", on the development of this "in-between orthorexia" profile.

Moreover, according to the results from ANOVA comparisons, the "H-IBO" cluster displayed greater levels of stress, depression and anxiety compared to other groups; as well as lower levels of self-esteem compared to “LO” cluster. As ON behavior has been suggested to represent a maladaptive eating behavior associated with depressive and anxiety symptoms [16], and low self-esteem [80, 81], this finding was not unexpected. It further suggests that the presence of high orthorexic tendencies in both their healthy and pathological forms can be concomitantly distressing. Therefore, this finding seems to support the clinical relevance of ON in regard to psychological consequences, albeit previous literature is not conclusive in this regard. Indeed, some evidence indicate that individuals with ON exhibit decreased levels of self-esteem [80, 81], as well as higher anxiety, depression, and emotional distress levels [82] compared to those with no orthorexic eating behaviors. Other studies, however, found that the variation in perceived stress and psychological well-being was no longer explained by ON when pathological eating was considered as a predictor [16]; which limits the evidence on causal link between ON and distress, and therefore the clinical significance of ON.

However, although significant, these results are quite negligeable in terms of effect size. Some explanations could account for these results. In fact, since ON has been suggested to be related to general psychopathology [11, 12, 14, 16, 23], one could have expected that our three distinct groups with low, moderate, and high degrees of ON, would also display important differences in terms of depression, anxiety, stress, and self-esteem. Indeed, previous studies have shown that variables such as depression and anxiety, which were observed at no or very low levels among individuals with no interest in healthy eating, increased significantly among individual with ON [49]. However, the reason why this difference remained negligible in the current study may be the variation in HO levels between the three clusters. In fact, many studies suggested that HO would represent a protective eating behavior, positively associated with positive affect [11] and adaptive personality traits (i.e., conscientiousness) [23], and negatively associated with overall psychopathology [1], health anxiety [22], stress or impulsivity [13]. Therefore, we cannot rule out the possibility of the co-occurrence of ON and HO reducing the negative effects of ON behavior to some degree.

Taken together, our findings are in line with and expand on previous studies suggesting orthorexic tendencies among adolescent populations [29, 32–34], and can further contribute to our understanding of adolescent vulnerability towards orthorexia. Indeed, our findings can help clinicians and researchers be aware of the existence of adolescents with an “in-between orthorexia” profile, engaging in a “healthy eating” behavior which has both healthy and pathological aspects [49]. If the potential confusion about the definition of the concept of "healthy eating" (which can, in part, account for the development of this orthorexia profile among adolescents), is taken into consideration and elucidated, we believe that this orthorexia profile may serve as a starting point in early prevention or treatment of ON behavior. More precisely, we argue that with the use of appropriate screening and intervention techniques (which also include psychoeducation on difference between ON and HO, as well as their associations and potential outcomes), the HO behavior which seem to reduce the negative effects of ON behavior to some degree can be enhanced, while the ON behavior can be prevented in early stages among young populations.

### Limitations

First, due to the recruitment of a non-clinical adolescent sample, findings from this study cannot be generalizable beyond Lebanese population, to clinical or older populations. It should also be noted that, many studies consider different age ranges for adolescence (e.g., between 10 and 19 years, as in [83] therefore our sample does not reflect the entire adolescence period. Secondly, the exploratory and cross-sectional nature of this study limits our ability to explain the causal interactions between study findings or whether the identified profiles are stable over time or whether individuals may switch from one group to the other. To overcome this issue, confirmatory techniques should be applied, and longitudinal studies should be conducted to assess whether observed profiles exist among different adolescent populations and whether they change over time. Future studies should also consider latent class analysis or latent class regressions as useful alternative techniques identify and distinguish between subgroups of individuals with similar characteristic within a same population. In addition, the unknown response rate might predispose us to a selection bias. It should also be noted that the recruitment for the current study took place during the national lockdown due COVID-19 pandemic outbreak. As many studies almost unanimously reported, this period considerably impacted individuals’ eating habits [83–85]. Among these latter, some studies even reported a shift towards healthy eating habits such as consumption of homemade preparations and fresh food, increased concern regarding food safety and hygienic practices [86], increased consumption of plants and pulses [84]. This could explain the presence of orthorexic tendencies among studied populations recruited during the lock-down period. Another study [85] found that the dietary pattern of adolescents during the COVID-19 pandemic lockdown was characterized by the consumption of both healthier (fish, meat, vegetables, and legumes) and non-healthier foods (e.g., alcohol and pastries). This could explain in part, our findings on the existence of an “in-between orthorexia” profile among adolescents. It is important to note that previous research also pointed out that several socio-demographic variables such as time spent together in family, experienced social isolation during pandemic [87] could also play a role in dietary changes of children and adolescents. Therefore, future studies should also consider these factors when exploring the profile of adolescents based on their orthorexic eating behaviors.

## Conclusion

Despite its limitations, this study is the first to explore the typology of adolescents based on ON and HO behaviors. Our findings expand the literature on profile and behavioral patterns of individuals with orthorexic eating behavior as they suggest the existence of a “In-between orthorexia” profile in adolescents. These individuals seem to engage in a “healthy eating” behavior which has both healthy and pathological aspects. Our findings on significant but negligible between group differences regarding several important psychological and psychopathological features further support the protective nature of HO behavior and highlight the possibility of the co-occurrence of ON and HO reducing the negative effects of ON behavior to some degree. Future studies should consider employing confirmatory and longitudinal approaches to explore whether identified orthorexia profiles are stable over time or whether individuals may switch from one group to another. Investigating whether several family-related (e.g., parental education, family food choices and eating habits, etc.) or other social factors (e.g., social media use, exposure to dietary or health-related content, etc.) may contribute to the development of (healthy or pathological) orthorexic behaviors can deepen our understanding of vulnerability towards orthorexia among young populations. The potential role of confusion around what constitutes "healthy eating" in the emergence of these "in-between orthorexia" profiles is put forward.

## Data Availability

All data generated or analyzed during this study are not publicly available to maintain the privacy of the individuals’ identities. The dataset supporting the conclusions is available upon request to the corresponding author.
